# “*Autonomy & Management in Prison Nursing*”. Development and validation of a mobile application

**DOI:** 10.17533/udea.iee.v44n1e09

**Published:** 2026-03-30

**Authors:** Leslie Diniz Alves, Leonardo Vieira Guimarães, Luiz Almeida da Silva, Maria Lucia do Carmo Cruz Robazzi, Namie Okino Savada, Fábio de Souza Terra, Sérgio Valverde Marques dos Santos

**Affiliations:** 1 Nurse, MSc, Email: leslie.diniz2015@gmail.com https://orcid.org/0000-0008-2431-9012 Universidade de São Paulo Brazil leslie.diniz2015@gmail.com; 2 Programmer. MSc. Email: leonardovieiraxy@hotmail.com https://orcid.org/0009-0000-3118-4664 Centro Universitário Leonardo Da Vinci Brazil leonardovieiraxy@hotmail.com; 3 Nurse, Ph.D. Email: enfer_luiz@ufcat.edu.br https://orcid.org/0000-0002-6661-035X enfer_luiz@ufcat.edu.br; 4 Nurse, Ph.D. Email: avrmlccr@eerp.usp.br https://orcid.org/0000-0003-2364-5787 Universidade de São Paulo Brazil avrmlccr@eerp.usp.br; 5 Nurse, Ph.D. Email: namie.sawada@unifal-mg.edu.br https://orcid.org/0000-0002-1874-3481 Universidade Federal de Alfenas Brazil namie.sawada@unifal-mg.edu.br; 6 Nurse, Ph.D. Email: fabio.terra@unifal-mg.edu.br https://orcid.org/0000-0001-8322-3039 Universidade Federal de Alfenas Brazil fabio.terra@unifal-mg.edu.br; 7 Nurse, Ph.D. Email: sergio.valverde@unifal-mg.edu.br. Corresponding author. https://orcid.org/0000-0001-8322-3039 Universidade de São Paulo Brazil sergio.valverde@unifal-mg.edu.br; 8 University of São Paulo, Ribeirão Preto School of Nursing - USP, Brazil. Universidade de São Paulo University of São Paulo Ribeirão Preto School of Nursing - USP Brazil; 9 Federal University of Alfenas, School of Nursing, Brazil. Universidade Federal de Alfenas Federal University of Alfenas School of Nursing Brazil; 10 Federal University of Catalão, Brazil. Federal University of Catalão Brazil; 11 Leonardo da Vinci University Center, Brazil. Centro Universitário Leonardo Da Vinci Leonardo da Vinci University Center Brazil

**Keywords:** correctional facilities, nursing care, technology and innovation, occupational health, psychometrics, reproducibility of results, mobile applications., instalaciones correccionales, atención de enfermería, tecnología e innovación, salud laboral, psicometría, reproducibilidad de resultados, aplicaciones móviles., estabelecimento penal, cuidados de enfermagem, tecnologia e inovação, saúde do trabalhador, psicometria, reprodutibilidade dos resultados, aplicações móveis.

## Abstract

**Objective.:**

To describe the process of development and validation of the content and semantics of the ***“Autonomy & Management in Prison Nursing”*** application for nurses working in prison units in Minas Gerais, Brazil.

**Methodology.:**

This methodological study employed the Contextualized Instructional Design method, following its four recommended stages: analysis, design and development, implementation, and evaluation. In the first phase, 164 nursing professionals participated by completing a semi-structured questionnaire addressing working conditions and professional autonomy. In the second phase, the application was developed with the support of an information technology professional. The third phase consisted of configuring and making the application available. Finally, in the fourth phase, content validation was carried out by 23 specialized judges and semantic validation by 51 nurses.

**Results.:**

The content validation of the application was satisfactory, as all indicators - total agreement rate, Content Validity Index (0.962; *p*<0.01), and overall Cronbach’s Alpha coefficient (0.969) - reached satisfactory levels. Semantic validation was also satisfactory, as all Cronbach’s Alpha coefficients achieved high values, indicating correlation among the items and excellent reliability of the application.

**Conclusion.:**

The *“Autonomy & Management in Prison Nursing”* application was developed and demonstrated satisfactory evaluation regarding both content and semantics, showing good adaptation to the proposed criteria and high internal consistency among the items.

## Resumen

**Objetivo.:**

Describir el proceso de desarrollo y validación del contenido y la semántica de la aplicación *“Autonomía & Gestión en enfermería penitenciaria”* para enfermeros que trabajan en unidades penitenciarias en Minas Gerais, Brasil.

**Métodos.:**

Estudio metodológico que empleó el método de Diseño Instruccional Contextualizado. Se siguieron los cuatro pasos recomendados: análisis, diseño y desarrollo, implementación y evaluación. La primera fase contó con la participación de 164 profesionales de enfermería que completaron un cuestionario semiestructurado con preguntas sobre las condiciones laborales y la autonomía profesional. En la segunda fase, se desarrolló la aplicación con el apoyo del profesional de tecnología de la información. La tercera fase consiste en la configuración de la aplicación y su puesta a disposición. Por último, en la cuarta fase, se realizó la validación de contenido por 23 jueces especialistas y de semántica por 51 enfermeros.

**Resultados.:**

La validación de contenido de la aplicación fue satisfactoria debido a que todos los resultados, el grado de concordancia total, el Índice de Validez de Contenido (0.962; p<0.01) y el coeficiente total del Alfa de Cronbach (0.969) alcanzaron valores considerados satisfactorios. La validación semántica también fue satisfactoria, ya que todos los resultados del coeficiente Alfa de Cronbach alcanzaron valores considerados altos, indicando correlación entre los ítems y excelente confiabilidad de la aplicación.

**Conclusión.:**

Se elaboró la aplicación denominada “*Autonomía & Gestión en Enfermería Penitenciaria”*, la cual tuvo una evaluación satisfactoria en relación con el contenido y la semántica, mostrando una buena adaptación respecto a los aspectos presentados y una alta consistencia interna entre los ítems.

## Resumo

**Objetivo.:**

Descrever o processo de desenvolvimento e validação de conteúdo e semântica do aplicativo *“Autonomia & Gestão em Enfermagem Prisional”* para enfermeiros atuantes em unidades prisionais em Minas Gerais.

**Métodos.:**

Estudo metodológico que empregou o método de Design Instrucional Contextualizado. Foram seguidos os quatro passos recomendados: análise, *design* e o desenvolvimento, implementação e avaliação. A primeira fase teve a participação de 164 profissionais de enfermagem que preencheram um questionário semiestruturado com questões relativas às condições laborais e autonomia profissional. Na segunda fase, foi desenvolvido o aplicativo com apoio do profissional de tecnologia da informação. A terceira fase constitui-se na configuração do aplicativo e disponibilização. Por fim, na quarta fase, foi realizada a validação de conteúdo por 23 juízes especialistas e semântica por 51 enfermeiros.

**Resultados.:**

A validação de conteúdo do aplicativo foi satisfatória devido ao fato de que todos os resultados, grau de concordância total, Índice de Validade de Conteúdo (0.962; *p*<0.01) e coeficiente total do Alfa de *Cronbach* (0.969) atingiram valores considerados satisfatórios. A validação semântica também foi satisfatória uma vez que todos os resultados do coeficiente Alfa de *Cronbach*, atingiram valores considerados altos, indicando correlação entre os itens e excelente confiabilidade do aplicativo.

**Conclusão.:**

Foi elaborado o aplicativo denominado *Autonomia & Gestão em Enfermagem Prisional,* o qual apresentou avaliação satisfatória em relação ao conteúdo e a semântica, possuindo uma boa adaptação quanto aos quesitos apresentados e alta consistência interna entre os itens.

## Introduction

The global situation of the prison system is marked by overcrowding, structural precariousness, human rights violations, and social inequalities that are reflected and exacerbated within prisons. In Brazil, it is no different; by operating far beyond its capacity (61.3% over), it creates a scenario that frequently denies inmates adequate access to humanization, rehabilitation, and, fundamentally, health services. This situation creates an inhumane and vulnerable environment, affecting both individuals deprived of their freedom and prison managers.^1^ In addition to overcrowding, the inadequate physical infrastructure, often composed of old police stations that have been adapted, worsens the problem. This results in places with poor ventilation and lighting, making them unhealthy and promoting the spread of diseases. The high prevalence of infections such as hepatitis B, tuberculosis, and syphilis in prisons elevates these establishments to the status of a serious public health problem.^1^^-^^3^

According to the Federal Constitution, in its Article 196, health is a right of all and a duty of the State. In other words, regardless of incarceration, the right to health is guaranteed through social and economic policies with universal and equal access. This right is highlighted in the Penal Execution Law nº 7.210, dated July 11, 1984.^2^ Despite the existence of the National Policy for Comprehensive Health Care for People Deprived of Freedom in the Prison System (PNAISP, as per its Portuguese acronym), which advocates care through a multidisciplinary team, the implementation of health care to inmates faces significant challenges. The disparity between installed capacity and the reality of services, the availability and training of health teams, and the complex integration of prison units with the SUS hierarchy are the main obstacles to the implementation of an health care program.^2^

Health care for the prison population often adopts a curative model, prioritizing emergency demands or specialized consultations, with care mediated by prison guards for security reasons. This momentum creates a dilemma for health professionals, who find themselves torn between their duty of care and institutional security requirements. Consequently, nurses and other professionals are not always able to provide quality care, as working conditions in prisons frequently direct primary attention to security rather than to the health needs of inmates.^1^ In this context, health care professionals, such as nurses working in the prison system, end up dealing with needs that differ from their usual practices, in a space marked by tension, with local limitations and characteristics unique to the prison environment.^3^ In this environment, it is the security professionals who are in direct contact with individuals deprived of their freedom and who end up hearing their complaints first and deciding whether they will be referred for care or if they will communicate the situation to the health professionals. This represents a limitation of nursing autonomy.^2^ As a result, nursing team professionals find themselves facing a two-way street, having to choose between their duty to provide care and their obligation to comply with the security regulations of these institutions.^1^

This situation is also found in other countries. A recent study evaluated the nursing practice environment in prisons in Portugal, aiming to strengthen human resources, infrastructure, and institutional policies to improve the quality of care and the well-being of professionals. The study highlighted the need for increased staff, better equipment and infrastructure, implementation of computerized information systems, greater recognition of the nursing role by management, and enhanced professional qualification. This demonstrates a continuous effort to optimize working conditions and improve the performance of nurses in Portugal’s prisons.^4^ Therefore, the development of technical-scientific knowledge, as well as the acquisition of new skills, is conducive to the change of habits, which can enhance workers’ health, reduce risky behaviors, and empower professionals to face the challenges of daily work life.^5^ Accordingly, the use of technology can contribute to the expansion of best practices and the improvement of care quality. An application can be an important tool in terms of improving the quality of management and care. Its importance lies in the ability to optimize crucial processes, from quick access to clinical protocols and updated guidelines, which minimize errors and standardize practices, to the detailed and secure recording of care, thus ensuring traceability and continuity of care. In practice, an application can empower nurses with information and resources that promote their professional autonomy, optimize the time spent on direct care, and facilitate qualified decision-making, resulting in more secure and efficient nursing services for the prison population.^5^^,^^6^

The increasing use of applications in the health field demonstrates the potential of technology to optimize processes and improve quality of life. The ability to search, organize, and interpret information efficiently is a skill that is increasingly valued, especially in terms of nursing, as it combines speed and accuracy, thus strengthening decision-making.^6^ Considering nursing professionals and the improvement and management of health care, the use of technology can be extremely important in ensuring health care actions; however, its implementation is a complex and dynamic process that requires collaboration among various stakeholders and the overcoming of challenges such as the lack of development of local technologies and bureaucratic barriers.^7^

Nursing plays a crucial role in promoting health within the prison system, an environment that, in Brazil, lacks technologies focused on managing the quality of health care services provided by these professionals. Currently, technological innovations in this context are mostly directed at the security and monitoring of inmates, as emphasized by studies.^5^^,^^6^ This gap in the application of technology for prison health management represents a significant challenge for qualified decision-making by nurses. Accordingly, this research is justified by its scientific and social relevance, as it seeks to fill this gap through the development and validation of an application. Therefore, the objective of this study was to describe the process of development and validation of the *Autonomia & Gestão em Enfermagem Prisional* application, a digital tool designed to assist nurses working in prisons in the state of Minas Gerais, promoting their professional autonomy and directly contributing to the qualified management of health care services in this complex environment.

Nursing plays a crucial role in promoting health within the prison system, an environment that, in Brazil, lacks technologies focused on managing the quality of health care services provided by these professionals. Currently, technological innovations in this context are mostly directed at the security and monitoring of inmates, as emphasized by studies.^5^^,^^6^ This gap in the application of technology for prison health management represents a significant challenge for qualified decision-making by nurses. Accordingly, this research is justified by its scientific and social relevance, as it seeks to fill this gap through the development and validation of an application - a need that is also observed in the international scenario, where there is a shortage of established models to support nursing practice in prisons. In this regard, the Brazilian development shown in this study provides a conceptual and methodological basis that can directly contribute to similar initiatives in other countries, such as Colombia, strengthening the production of health technologies aimed at the prison context. Therefore, the objective of this study was to describe the process of development and content and semantic validation of the *Autonomia & Gestão em Enfermagem Prisional* application for nurses working in prison units in Minas Gerais, Brazil.

## Methods

This is a methodological study, using the Contextualized Instructional Design (CID) method,^8^ which involved the development and validation of content and semantics of an application to optimize the work and strengthen the autonomy of nursing professionals in the prison system. CID is a methodological framework that values human activity and balances technological resources and creative processes, guiding the planning, development, and application of content through Information and Communication Technologies, with an emphasis on contextualization and flexibility.^8^

The main functionality of this application is to provide quick and updated access to crucial information, such as protocols and guidelines, as well as to enable efficient management of records and resources, directly impacting the improvement of the quality of care and actions provided to individuals deprived of freedom. Data collection took place from July 2023 to April 2024. In order to carry out the development and validation of the application content and semantics for mobile devices, the four steps recommended by the CID method were followed, namely: analysis, design and development, implementation, and evaluation. 

The first phase, the content analysis, consisted of evaluating the results obtained in the survey of working conditions and autonomy, conducted through a descriptive analysis with 164 nursing professionals from the prison system of Minas Gerais, Brazil, using a semi-structured questionnaire based on the researchers’ experiences, with the objective of evaluating sociodemographic and job characteristics, working conditions, and autonomy. This instrument included variables such as: sex, age, marital status, number of children, family income, type of housing, alcohol consumption, cigarette consumption, physical activity, chronic disease, continuous use of medication, professional category, length of nursing career, time of nursing practice in the prison institution under study, workload, work period/shift, activity sector, and whether they had another job. At this stage, the inclusion criterion was to be working in nursing in the prison units of Minas Gerais during the period when data collection took place. 

Based on these results, the main axes were defined for the preparation of support materials for nursing professionals, aiming to enhance the management of health care in the prison system. These axes included essential aspects of practice, such as nursing legislation, work standards and routines, standard operating procedures (SOPs), prison system legislation, and health protocols applicable to the prison context. The research also included the analysis of official documents and relevant references, such as the Regulation of Nursing Practice, the Implementation of the Nursing Process in Socio-Environmental Contexts,^9^ the Guidelines for Nursing Team Performance in Primary Health Care,^10^ the Nursing Service Regulations in the Prison Units of Minas Gerais,^11^ the Nursing Process Applied to Incarcerated Individuals,^12^ the National Policy for Comprehensive Health Care for People Deprived of Freedom in the Prison System (PNAISP),^13^ and the Standard Operating Procedures Handbook for Nursing.^14^ Subsequently, a survey was conducted on the main diseases affecting this population through consultations on the Minas Gerais Penal Police website and internet searches using the terms: professional autonomy, prison system, person deprived of freedom, diseases, and primary health care.

The second phase, design and development, was carried out by the Information and Communication Technology (ICT) professional, based on the text materials and figures offered by the researchers, providing greater detail to the technological tool. The application was developed with a focus on human-computer interaction, employing computational tools and the React Native framework, together with Expo.^15^ The source code of this project was made available on GitHub, providing transparency and collaboration. Accordingly, the application was titled: *Autonomia & Gestão em Enfermagem no Sistema Prisional* (*A&G Enfermagem Prisional*), which can be translated as Autonomy & Nursing Management in the Prison System (A&G Prison Nursing).

In the third phase, implementation, the application was configured and the prototype was made available for download on the GitHub platform, which is a cloud-based Git repository hosting service that allows individuals and teams to use Git in a simplified way for version control and collaboration.^16^ The *A&G em Enfermagem Prisional* application is available for Android via the following QR Code:

**Figure:**
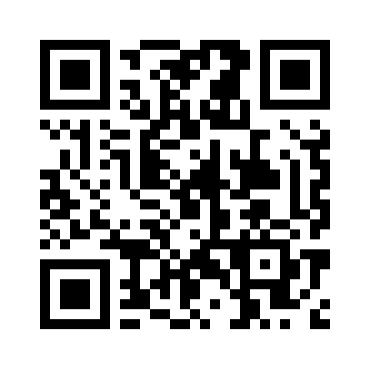

The fourth phase, evaluation, was divided into two stages: content validation and semantics validation. Content validation was a stage focused on a thorough evaluation of the content of an instrument, based on the verification of items using criteria that could ensure quality and validate the adequacy of the proposal.^17^ In this stage, 23 judges from the Nursing field participated in the study. These specialists met the following inclusion criteria: holding at least a doctoral degree, being active as a university professor, and having expertise in the topic under study. A Likert-type instrument was used in this evaluation to check the variables: appearance, relevance, understanding, functionality, and information of the application. This instrument was developed by the authors, based on other studies related to technological content validation.^15^^,^^17^^-^^19^

In order to evaluate the internal consistency of the instrument, the Cronbach’s Alpha coefficient was used, considering an interval between 0.70 and 0.95 as adequate.^20^ Agreement among the judges was analyzed using the Content Validity Index (CVI), a psychometric measure that represents the proportion of agreement on the evaluated items.^18^ CVI values per item ≥0.78 and an overall CVI average >0.90 were adopted as the acceptability criteria.^19^ In order to evaluate the suitability of each item to the psychometric criteria, the average of each parameter was calculated by dividing the total sum of scores by the number of judges. In order to estimate the statistical reliability of the CVI indexes, the exact test of the binomial distribution was used, which is recommended for small samples. A significance level of p<0.05 and a minimum agreement proportion of 0.95 were considered. In this test, the null hypothesis was: the application did not have good adaptation, and the alternative hypothesis was that the evaluators considered the adaptation of this application to the proposed criteria as adequate.

The second stage of the evaluation, semantic validation, consisted of evaluating the clarity of the items, ease of reading, understanding, and their presentation format. This stage was carried out by a group of 51 nursing professionals, selected according to the following inclusion criteria: having participated in the first stage and currently working in the prison system of Minas Gerais at the time of the research. Semantic validation makes it possible to verify whether the items are understandable to the study population and allows the instrument to be adapted so that it is easy to understand and has quality and coherence for the target audience. It identifies problems related to the acceptance and judgment of the items included in the proposed instrument by the research subjects.^21^^,^^22^

For this stage, a Mobile Application Rating Scale (uMARS) was used, which was created and validated in Brazil by Gralha and Bittencourt in 2023 to evaluate the quality and content of applications related to the health field. It is a multidimensional instrument, recommended for use by specialists, to evaluate the quality of health applications.^23^ It had 20 items included in five subscales in total, being four subscales of objective quality - engagement, functionality, esthetic, and information quality - and one subscale of subjective quality, which includes six items and is added to measure the perceived impact on users of the application being evaluated. In this stage of the study, the Cronbach’s Alpha coefficient was used to analyze the semantic validity of the content of this application, with the aim of evaluating the internal consistency of the items and whether the data were correlated with each other.^24^ In this analysis, a coefficient above 0.70 was considered adequate.^25^ For all analyses, the Statistical Package for the Social Sciences (SPSS) program, version 17.0, was used. For all analyses, a 95% confidence interval and 5% significance level were considered. 

This study was approved by the Research Ethics Committee of the State University of Minas Gerais, Brazil, with Opinion nº 6.003.311 and CAAE nº 67084223.3.0000.5112. All participants signed the Free and Informed Consent Form.

## Results

In the evaluation of the survey on working conditions and autonomy of nursing professionals in the prison system according to characterization variables, it was observed that there was a higher frequency of female participants (81.7%), married individuals (50%), and those aged between 30 and 39 (47.6%). Regarding job issues related to nursing professionals, most had permanent employment (66.5%), were nurses (56.7%), worked 4x1 shifts (62.5%), during the daytime (50.6%), with a daily workload of 9 to 11 hours (43.3%) and a weekly workload of up to 40 hours (97.6%). More than half of the professionals had between 11 and 20 years of experience in nursing (56.7%), and 79% had up to 10 years of service at the institution.

Regarding the workplace, 90.2% and 64.6%, respectively, considered that they had space and materials for work, and 90.2% did not have a place to rest. It was also observed that most were not trained for the job (85.4%), 86.0% reported that the job has occupational hazards, and 87.8% stated that they had Personal Protective Equipment. These workers (73.2%) stated they had average professional autonomy. The analyses of the content validation of the application conducted by the judges are displayed in Table 1.

**Table 1:** Content evaluation of the *Autonomia & Gestão em Enfermagem no Sistema Prisional* application according to the Content Validity Index, based on the responses of 23 judges. MG, Brazil, 2023

Application items	CVI-i	***p*-value**	Cronbach’s Alpha
Home Page	0.957	<0.001	0.125
Legislation	0.957	<0.001	0.583
RENP*- Legislation	0.965	<0.001	0.713
LEP**- Legislation	0.957	<0.001	0.767
Professional Practice Law - Legislation	0.957	<0.001	0.767
PNAISP***- Legislation	0.965	<0.001	0.767
Workload Resolution - Legislation	0.965	<0.001	0.767
Standards and Routines	0.965	<0.001	0.699
Nursing Regulations - Standards and Routines	0.957	<0.001	0.713
SOP - Standards and Routines	0.957	<0.001	0.767
Transportation Handbook - Standards and Routines	0.956	<0.001	0.767
Primary Care Guide - Standards and Routines	0.957	<0.001	0.852
Health Protocol	0.957	<0.001	0.625
Tuberculosis - Health Protocol	0.965	<0.001	0.366
STI/AIDS - Health Protocol	0.948	<0.001	0.645
Scabies - Health Protocol	0.939	<0.001	0.770
Level of autonomy	0.991	<0.001	-
Nursing activity scale	0.983	<0.001	-
Talk to DSP	0.991	<0.001	-
About the app	0.974	<0.001	0.625
Total CVI	0.962		
Total Cronbach’s Alpha			0.969

*Regulations and Procedural Rules of the Prison System of Minas Gerais; **Penal Execution Law; ***National Policy for Comprehensive Health Care for People Deprived of Freedom

In the analysis of the items and their adequacy to the criteria, an average was obtained for each parameter for the entire indicator, divided by the number of judges. Accordingly, the exact binomial distribution test showed that the agreement index among the judges was significant, p<0.001 for all items, indicating that the application has good adaptation regarding the introduced criteria. In the analysis of internal consistency, using Cronbach’s Alpha, it was observed that most items scored above 0.70, as recommended, with a total Alpha coefficient of 0.969, representing a high internal consistency among the items. Given the above, the results displayed in Table 1 show that the content validation of the application carried out by the judges was satisfactory. This is due to the fact that all the results, that is, the total degree of agreement, the CVI, the significant p-value, and the total Cronbach’s Alpha coefficient, reached values considered high. The analyses of the semantic validation of this application carried out by nursing professionals are displayed in Table 2.

**Table 2:** Evaluation of the application semantics using the Cronbach’s Alpha coefficient according to the reliability of the responses for each uMARS domain* by 51 nursing professionals. MG, Brazil, 2023

Domains	Total	Average	SD	Cronbach’s Alpha
Engagement				
Entertainment	194	3.80	1.08	0.874
Interest	198	3.88	1.09	
Customization	144	2.82	1.47	
Interactivity	153	3.00	1.25	
Target Audience	215	4.22	0.94	
Functionality				
Performance	201	3.94	1.05	0.875
Easy to Use	212	4.16	0.95	
Browsing	203	3.98	0.88	
Gestural Design	209	4.10	0.90	
Esthetic				
Layout	196	3.84	1.01	0.939
Graphics	187	3.67	1.05	
Visual Appeal	192	3.76	0.89	
Information				
Quality of Information	216	4.24	0.86	0.907
Quantity of Information	227	4.45	0.76	
Visual Information	230	4.51	0.81	
Credibility of Source	223	4.37	0.82	
Subjective Quality				
Recommendation	195	3.82	1.03	0.864
Usage	190	3.73	1.04	
Payment	136	2.67	1.35	
Stars	181	3.55	0.99	
Specific Health Evaluation				
Awareness	199	3.90	1.08	0.949
Knowledge	192	3.76	1.11	
Attitudes	194	3.80	1.13	
Intention to change	188	3.69	1.14	
Looking for help	192	3.76	1.16	
Behavior change	177	3.47	1.24

*uMars: Mobile Application Rating Scale

In Table 2, it was possible to evaluate the semantics of the *Autonomia & Gestão em Enfermagem no Sistema Prisional* application through internal consistency and the reliability index using the Cronbach’s Alpha coefficient. It was observed that, in all uMARS domains, the reliability index was higher than 0.70, that is, between 0.86 and 0.94, considered high internal consistency, showing homogeneity and reliability in its items. The last uMARS domain, regarding specific health evaluation, was the one that showed the highest internal consistency (0.949). The results displayed in Table 2 show that the semantic validation of the application carried out by nursing professionals was satisfactory. This is due to the fact that all the results of the Cronbach’s Alpha coefficient reached values considered high. These values indicate that there is a satisfactory correlation among the items and demonstrate that the application has high reliability. 

**Figure 1:**
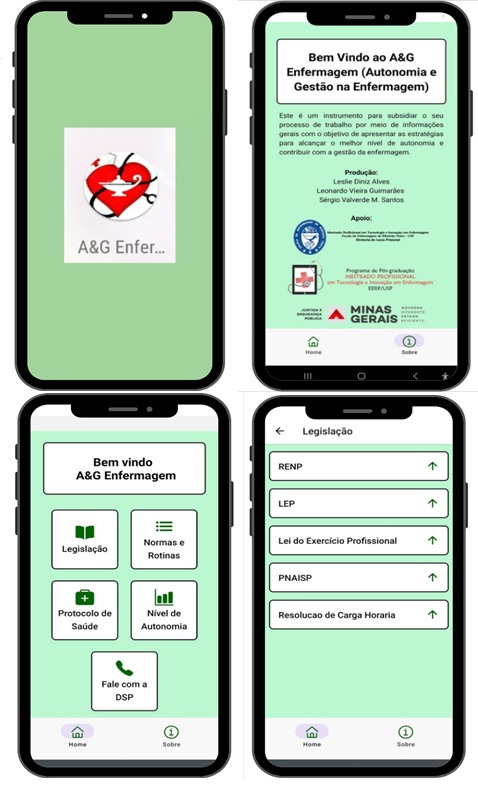
Main screens of the *Autonomia & Gestão em Enfermagem no Sistema Prisional* application

## Discussion

The *Autonomia & Gestão em Enfermagem no Sistema Prisional* application was created to support the nursing work process by providing general information from the technical area and aiming to introduce strategies focused on achieving the highest level of professional autonomy and contributing to work management. For a developed application to be used, it needs to be validated by specialists. Validation is important to evaluate the quality of the produced artifacts, being a crucial step in the development of the technological tool and to evaluate its appearance, content, consistency, and whether the requirements are reliable for practice and secure.^26^ The application underwent a content and semantic validation process, allowing the analysis of its applicability, quality, operability, and content. In the content validation process of this application, it was observed that the validation was satisfactory, achieving a CVI above 0.70 (ranging from 0.93 to 0.99), with an overall CVI average of 0.962. In addition, the exact binomial distribution test showed that the agreement index among the judges was significant (p<0.01) for all items, indicating that the application is well adapted regarding the evaluated aspects. Furthermore, the analysis of internal consistency, using Cronbach’s Alpha, showed a total alpha coefficient of 0.969, representing a high internal consistency among the items evaluated by the judges in the application.

The results of the content validation of this the application are similar to those by a study on the development of a mobile application for prenatal care monitoring in Brazil, which showed an adequate Content Validity Index (CVI), demonstrating that the addressed content and the technical aspects of the system were reliable and valid.^27^ The overall CVI of the content evaluation of an application for adolescents with diabetes mellitus was 0.93, which matched the result of this study. Furthermore, it was confirmed that the application has a simple, user-friendly, and pleasant interface, observing the principles of functionality, usefulness, and reliability, with easy and quick access, thus improving its efficiency.^28^ The semantic validation process of the application was satisfactory, since the reliability indexes revealed in all uMARS domains by the Cronbach’s Alpha coefficient was greater than 0.70 (between 0.86 and 0.94), considered high internal consistency, showing homogeneity and credibility in its items, indicating a satisfactory correlation among the items and demonstrating that the application has high reliability. 

A study on the semantic validation of a mobile application for the prevention and control of syphilis in Brazil showed results similar to this research, with a Cronbach’s Alpha coefficient of 0.848, suggesting good internal consistency among the judges.^29^ Similarly, a study conducted in the Philippines, regarding the detection of dengue outbreaks through an application, also using the uMARS instrument, showed an excellent level of consistency indicated by a Cronbach’s Alpha coefficient of 0.9.^30^ These results show that the use of technologies such as applications in nursing practice can help with access to information, make health care more organized, speed up and systematize the work process, optimize the professional’s time, and support the management of activities.^28^ Thus, validated health applications are a current alternative that can contribute to health education, both in its informative aspect and in the evaluation, diagnosis, and treatment of diseases, enabling improvements in the relationship between health care professionals and patients and eliminating inconsistencies in the provided information.^31^

The nursing performance in correctional environments in Brazil is still an emerging field of study, especially concerning professional autonomy. This gap points to an urgent need for more research focused on nursing practice in these workplaces. At the same time, it is observed that technological innovations in the prison system have been developed almost exclusively to increase the security of the environment, neglecting the development of technological methodologies that could optimize the performance of nursing professionals themselves in promoting the health of inmates. This technological gap was a significant limitation for the current study, making it difficult to compare results due to the shortage of data and related research. Another notable challenge was the difficulty in reaching the target audience of this research. Even with the issuance of formal documents by central authorities to facilitate access, many professionals did not come into contact with the data collection instruments or chose not to participate.

Conclusion. The *Autonomia & Gestão em Enfermagem no Sistema Prisional* application demonstrated adequate content and semantic validity, as well as high internal consistency, proving to be a reliable resource to support nursing practice in the prison system. Its adoption can strengthen professional autonomy, standardize procedures, and enhance the management of health care for people deprived of freedom by providing updated and easily accessible information. For practice, the application can contribute to quicker and more informed decisions. In education, it can assist in the training of students and professionals by integrating digital technologies into the educational process. For research, it is recommended to conduct implementation studies and impact evaluations in order to measure its effects on work organization and health outcomes in the prison context.
